# Donor-Derived Testicular Germ Cell Cancer in a Heart Transplant Recipient

**DOI:** 10.1016/j.jaccao.2021.02.009

**Published:** 2021-06-15

**Authors:** Marish I.F.J. Oerlemans, Gerard Groenewegen, Aryan Vink, Linda W. van Laake, Niels P. van der Kaaij, Nicolaas de Jonge

**Affiliations:** a Department of Cardiology, University Medical Center Utrecht, Utrecht, the Netherlands; b Department of Medical Oncology, University Medical Center Utrecht, Utrecht, the Netherlands; c Department of Pathology, University Medical Center Utrecht, Utrecht, the Netherlands; d Department of Cardio-Thoracic Surgery, University Medical Center Utrecht, Utrecht, the Netherlands

**Keywords:** cardiac masses, diagnosis, germ cell tumors, heart failure, treatment, αFP, alfa-fetoprotein, CT, computed tomography, hCG, human chorionic gonadotropin, HTx, heart transplantation, RV, right ventricle, TGCC, testicular germ cell cancer

Heart transplantation (HTx) is considered the gold standard therapy for selected patients with end-stage heart failure, significantly improving survival and quality of life in comparison to medical therapy (1). Necessary immunosuppression results in an increased risk of malignancy, but transmission of donor-derived solid tumors is rare due to stringent criteria in donor selection (2). Testicular germ cell cancer (TGCC) is a highly treatable solid neoplasm; high cure rates can be obtained with cytotoxic chemotherapy. However, metastases of TGCC to the heart are very rarely detected in clinical practice (3). Here, we present a case of *transplanted* germ cell cancer after HTx, detected by endomyocardial biopsy.

## Case Presentation

A 57-year-old male patient underwent aortic valve replacement because of severe aortic regurgitation at a referring hospital at 38 years of age. In the subsequent 9 years, he underwent 4 reoperations because of recurrent endocarditis. After progressive decline of his left ventricular (LV) function, he was transferred to our center and listed for HTx.

A donor heart became available of a young man in his mid-twenties, presumed previously healthy. He was diagnosed with pulmonary embolism after a long-distance flight, which led to an out-of-hospital cardiac arrest with pulseless electrical activity. Echocardiography showed a severely dilated right ventricle (RV) with poor function and LV cavitary obliteration. After resuscitation, he was hemodynamically stable and transported to the intensive care unit. Unfortunately, he was severely neurologically damaged, and brain death was diagnosed after 2 days. Based on serial echocardiography that showed a recovered RV, the heart was accepted for donation and a donor procedure was started.

Donor heart procurement was uneventful. Because of the 5 previous sternotomies and a high likelihood of a complicated recipient cardiectomy, to limit cold ischemia time, the recipient operation was started as soon as the heart was accepted at the donor site. Upon arrival to our hospital, it became apparent that a cancerous process was present in the retroperitoneum of the donor, which had not been recognized earlier by conventional imaging and inspection. Pathology of this process showed a mixed germ cell cancer (embryonal carcinoma with component of yolk sac tumor). Donor post-procedure blood results showed elevated values for both alfa-fetoprotein (αFP) and human chorionic gonadotropin (hCG). The diagnosis of metastatic testicular germ cell cancer (TGCC) in the donor was made.

Unfortunately, the diagnosis of metastatic TGCC came after the recipient surgical team had already started cardiectomy of the recipient, reaching a point of no return. Although any possibility of cancer transmission to a transplant recipient should be avoided, it was decided that there was no other option than to continue with the transplantation. An oncologist specializing in TGCC was consulted. The risk of poor outcome with venoarterial extracorporeal membrane oxygenation and waiting for an urgent donor heart was thought to be greater than the risk of transmission of TGCC, given the significant shortage of donor hearts and relatively high waiting list mortality. Additional irrigation of the heart was performed to remove as many intravascular cells as possible. Implantation of the donor heart was uneventful. Immunosuppression was started as usual—tacrolimus, mycophenolate mofetil (MMF), and prednisolone—in combination with increased oncological surveillance.

Temporary venoarterial extracorporeal membrane oxygenation support because of RV failure and hemofiltration was necessary for 3 days. The patient was extubated on day 6 and then transferred to the cardiology ward on day 9 post-operatively. He recovered quickly and had normal cardiac function and rhythm. Routine endomyocardial biopsy on days 16 and 23 showed 1R cellular rejection, for which prednisolone tapering was deferred, and he remained at a dose of 20 mg/day. Thereafter, endomyocardial biopsy normalized and surveillance for clinical stage 1 TGCC was initiated by the oncologist. Two months after HTx, serum tumor markers (αFP and hCG) were normal. Computed tomography (CT) of the chest and abdomen did not show any metastases, and ultrasound of the testes was normal as well.

**Figure 1:**
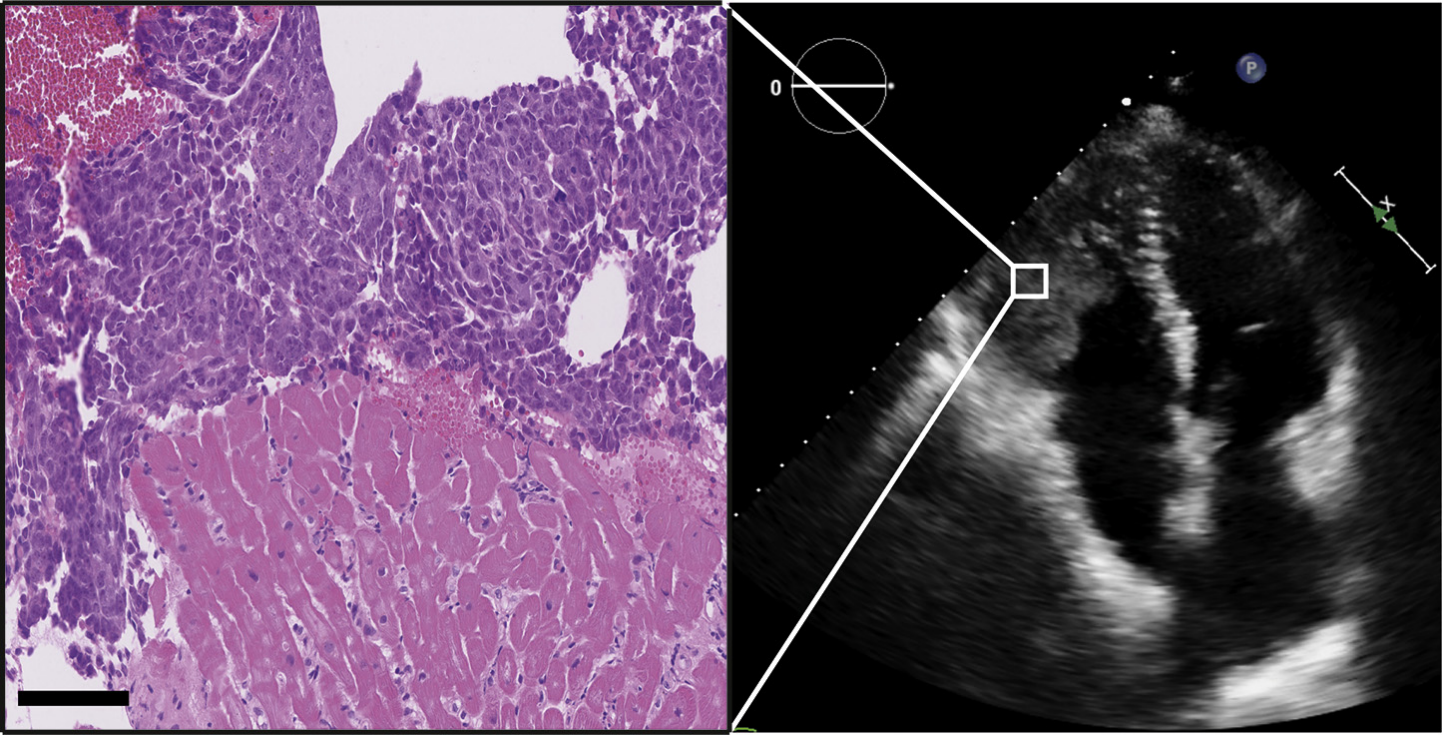
Endomyocardial Biopsy and Echocardiographic Imaging at 3-Month Follow-Up Echocardiographic imaging of the right ventricular mass in the apical 4-chamber view **(right)**. Hematoxylin and eosin staining of the endomyocardial biopsy showed normal myocardium without signs of rejection **(left)**. On top, proliferation of cells with cytonuclear atypia, which were positive for CD30, CK AE1/3, OCT3/4, and PLAP immunostains, compatible with a germ cell tumor subtype embryonal carcinoma. Bar = 100 μm.

**Figure 2:**
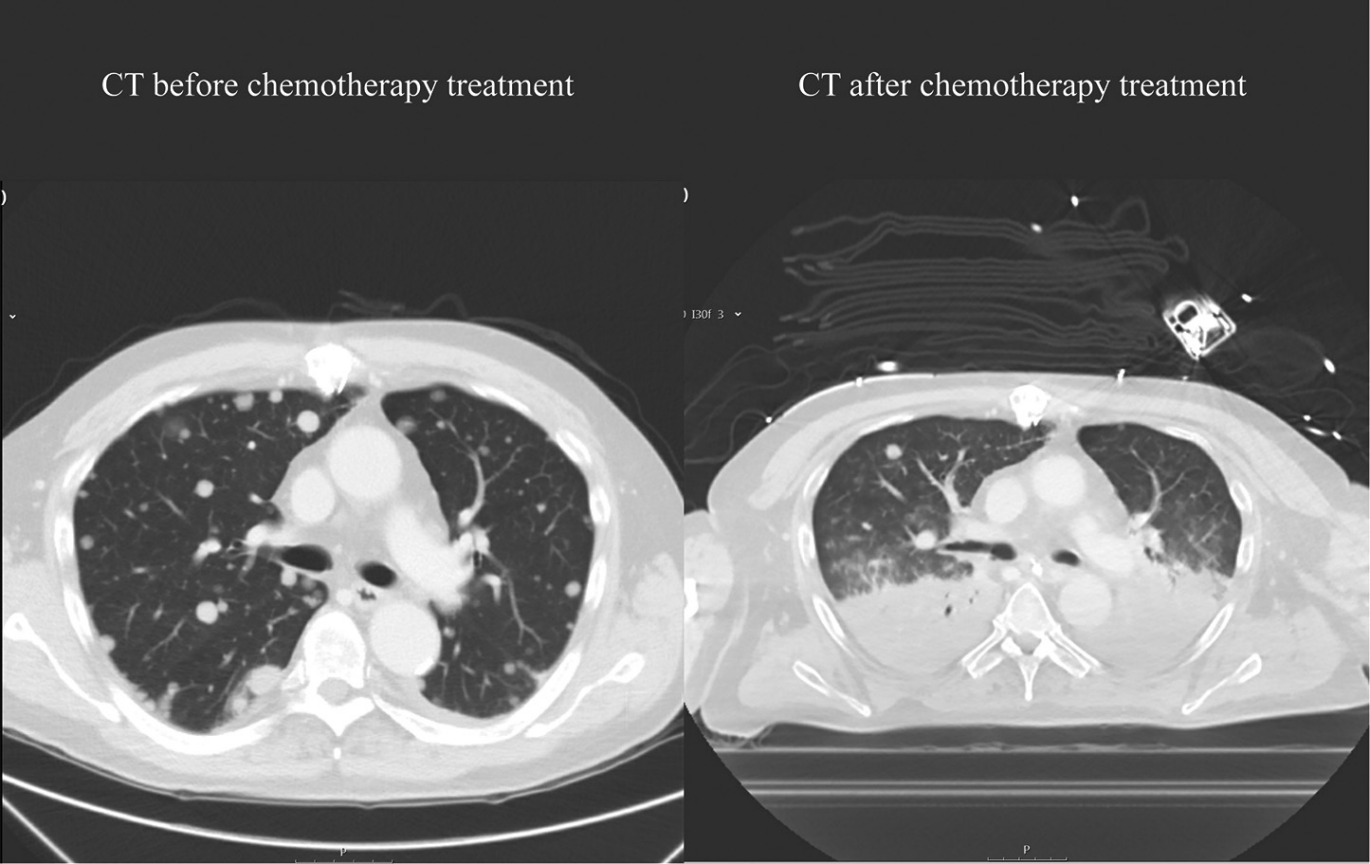
Chest CT Scan Before and After Initiation of Chemotherapy Chest computed tomography (CT) scan before chemotherapy showing extensive pulmonary metastases with enlarged mediastinal lymph nodes **(left)**. On the day 11 after chemotherapy initiation, a chest CT post-cardiac arrest showed bilateral infiltrates in the lungs and regression of pulmonary metastases **(right)**.

Three months after HTx, pathological examination of a routine endomyocardial biopsy showed embryonal carcinoma, without signs of rejection of the heart or cellular infiltrate in the tumor (Figure 1, left panel). Echocardiography showed a large intracardiac mass in the RV without inflow or outflow obstruction (Figure 1, right panel). Restaging of TGCC was performed only 3 weeks after the previous surveillance measurement, now showing elevated αFP (25 μg/l), hCG (30 IU/l), and LDH (4× upper limit of normal). CT scan showed multiple pulmonary metastases, only in the lungs without other localizations (Figure 2, left panel). A diagnosis of transplanted metastatic germ cell cancer was made.

After discussions with patient, family, and experts in the field of TGCC, it was decided to start cytotoxic chemotherapy consisting of a 5-day regimen of cisplatin and etoposide. The dose of MMF was reduced to 250 mg twice daily, and target tacrolimus levels were reduced to 5 μg/l. Continuous heart rate monitoring during treatment did not show any abnormalities, and the patient was discharged on day 6 without signs of heart failure or other toxicities.

Ten days post-chemotherapy, he experienced diarrhea without fever, and loperamide was started. The next day, however, he arrested, and the first registered rhythm was asystole. After successful resuscitation, the patient was transported to a nearby hospital. Echocardiography showed a normal LV and RV function without pericardial effusion. A CT scan was performed, which showed bilateral infiltrates in the lungs and signs of colitis. The CT also showed regression of pulmonary metastases (Figure 2, right panel).

Following admission to the intensive care unit, broad spectrum antibiotics and vasopressors were given, but the patient deteriorated further and developed an acidosis with pH 6.7. The family withdrew treatment. The patient died at 58 years of age; an autopsy was not performed.

## Discussion

In this case, several clinical dilemmas are raised, which together have led to an unfortunate series of events. These included: a history of many previous sternotomies, the finding of cancer while the surgical procedure was beyond a critical stage, the rare case of donor-transmitted cancer, and the poor outcome despite regression of pulmonary metastases and the treatment of TGCC with curative intent.

A recent single-center analysis reported secondary cardiac metastatic tumors in 3 of 142 (2.1%) patients, and none with TGCC (4).Given the very low incidence of TGCC and stringent donor screening and selection, the risk of donor-transmitted malignancies was felt to be very low (2,3,5). Data on actual transmissions are sparse or restricted to data on incidence of all malignancies occurring after HTx, of which donors with central nervous system tumors and melanoma are mostly described in solid organ transplantation. A recent review on kidney transplant recipients of tumor-bearing donors reported a 1-year mortality of 50% in donor-derived melanoma (6). Therefore, donor malignancy is a major contraindication for HTx.

An important issue is the donor screening of the previously healthy young male with massive pulmonary embolism after a long-distance flight. Although not regarded as truly “unprovoked” in our case, occult cancer can be found in 5% of patients with unprovoked thromboembolism (7). In retrospect, it became clear that a CT of the chest only could not visualize the retroperitoneal mass in the donor work-up. We, therefore, perform a total body CT in all potential donors with pulmonary embolism, and we have a standardized necessity of a full report in our procedures.

The case also illustrates that TGCC can metastasize to the heart (8). The transmission via the heart resulted in metastases in the lungs, suggesting an important role of donor circulating tumor cells. Nowadays, excellent cure rates can be obtained with cytotoxic chemotherapy, even with metastatic TGCC (9). Despite the use of tacrolimus and MMF, regression of the pulmonary metastases was observed on CT*.* Although a switch to sirolimus was considered given its potentially antineoplastic properties, 2 important arguments were raised against its use: 1) its efficacy in TGCC is questionable (10); and 2) sirolimus clearance is increased when dexamethasone (as an antiemetic drug) is added, leading to an unstable immunosuppressive state and unacceptable risk of graft rejection.

The presented patient died acutely of toxicities such as neutropenia and mucositis, but with a primary cause of death being the cardiac arrest and its sequelae. It can certainly not be excluded that necrosis in the cardiac tumor was the trigger for cardiac arrhythmias, although not observed during chemotherapy administration. In summary, we report the case of a patient in whom a heart and a potentially curable tumor were transplanted. Unfortunately, the recipient of each died.

## Funding Support and Author Disclosures

The authors have reported that they have no relationships relevant to the contents of this paper to disclose.
